# Trisodium citrate 4% versus heparin as a catheter lock for non-tunneled hemodialysis catheters in critically ill patients: a multicenter, randomized clinical trial

**DOI:** 10.1186/s13613-019-0553-4

**Published:** 2019-07-01

**Authors:** Jean-Pierre Quenot, Julie Helms, Abderrahmane Bourredjem, Auguste Dargent, Ferhat Meziani, Julio Badie, Gilles Blasco, Gaël Piton, Gilles Capellier, Chaouki Mezher, Jean-Michel Rebibou, Abdelouaid Nadji, Thomas Crepin, Saber Davide Barbar, Camille Fleck, Amélie Cransac, Mathieu Boulin, Christine Binquet, Agnès Soudry-Faure, Rémi Bruyère, for the VERROU-REA Trial Investigators and the CRICS TRIGGERSEP Group (Clinical Research in Intensive Care and Sepsis Trial Group for Global Evaluation and Research in Sepsis)

**Affiliations:** 1 Service de Médecine Intensive-Réanimation, Centre Hospitalier Universitaire Dijon Bourgogne, 14 rue Paul Gaffarel, B.P 77908, 21079 Dijon Cedex, France; 2 Université Bourgogne Franche-Comté, Lipness Team UMR 1231 et LabExLipSTIC, 21000 Dijon, France; 3 INSERM, CIC 1432, module Epidémiologie Clinique, 21000 Dijon, France; 4 CHU de Dijon, Centre d’Investigation Clinique, module Epidémiologie Clinique/Essais cliniques, 21000 Dijon, France; 5 CHRU de Strasbourg, Nouvel Hôpital Civil, Service de Réanimation Médicale, 67000 Strasbourg, France; 6 Université de Strasbourg, UMR 1260, régénérative nanomédicine, FMTS, 67 000 Strasbourg, France; 7 CH de Belfort-Montbéliard, Service de Réanimation Polyvalente, 90000 Belfort, France; 8 CHU de Besançon, Service de Réanimation Chirurgicale, 25000 Besançon, France; 9 CHU de Besançon, Service de Réanimation Médicale, 25000 Besançon, France; 10 Université de Franche-Comté, EA 3920, 25000 Besançon, France; 11 CH Belfort-Montbéliard, Service de Réanimation Polyvalente, 25200 Montbéliard, France; 12 CHU Dijon Bourgogne, Service de Néphrologie, 21000 Dijon, France; 13 CHU Dijon Bourgogne, Service de Réanimation Neuro-Traumatologique, 21000 Dijon, France; 14 CHU de Besançon, Service de Soins intensifs Néphrologie, 25000 Besançon, France; 15 CHU de Nîmes, Service de Réanimation Médicale, 30 000 Nîmes, France; 16 CHU Dijon Bourgogne, Délégation à la Recherche Clinique et à l’Innovation (DRCI), 21000 Dijon, France; 17 Département de Pharmacie, CHU Dijon Bourgogne, 21000 Dijon, France; 18 Université de Bourgogne Franche-Comté, LNC-UMR 1231, 21000 Dijon, France; 19 CHU Dijon Bourgogne, Unité de Soutien Méthodologique à la Recherche (USMR), 21000 Dijon, France; 20 CH de Bourg en Bresse, Service de Réanimation polyvalente, 01000 Bourg en Bresse, France

**Keywords:** Citra-Lock, Heparin, Catheters, Acute renal failure, Hemodialysis, Critical illness

## Abstract

**Background:**

Non-tunneled hemodialysis catheters are currently used for critically ill patients with acute kidney injury requiring extracorporeal renal replacement therapy. Strategies to prevent catheter dysfunction and infection with catheter locks remain controversial.

**Methods:**

In a multicenter, randomized, controlled, double-blind trial, we compared two strategies for catheter locking of non-tunneled hemodialysis catheters, namely trisodium citrate at 4% (intervention group) versus unfractionated heparin (control group), in patients aged 18 years or older admitted to the intensive care unit and in whom a first non-tunneled hemodialysis catheter was to be inserted by the jugular or femoral vein. The primary endpoint was length of event-free survival of the first non-tunneled hemodialysis catheter. Secondary endpoints were: rate of fibrinolysis, incidence of catheter dysfunction and incidence of catheter-related bloodstream infection (CRBSI), all per 1000 catheter-days; number of hemorrhagic events requiring transfusion, length of stay in intensive care and in hospital; 28-day mortality.

**Results:**

Overall, 396 randomized patients completed the trial: 199 in the citrate group and 197 in the heparin group. There was no significant difference in baseline characteristics between groups. The duration of event-free survival of the first non-tunneled hemodialysis catheter was not significantly different between groups: 7 days (IQR 3–10) in the citrate group and 5 days (IQR 3–11) in the heparin group (*p* = 0.51). Rates of catheter thrombosis, CRBSI, and adverse events were not statistically different between groups.

**Conclusions:**

In critically ill patients, there was no significant difference in the duration of event-free survival of the first non-tunneled hemodialysis catheter between trisodium citrate 4% and heparin as a locking solution. Catheter thrombosis, catheter-related infection, and adverse events were not statistically different between the two groups.

*Trial registration* Registered with Clinicaltrials.gov under the number NCT01962116. Registered 14 October 2013.

**Electronic supplementary material:**

The online version of this article (10.1186/s13613-019-0553-4) contains supplementary material, which is available to authorized users.

## Background

Non-tunneled hemodialysis catheters are currently the preferred vascular access method for critically ill patients with acute kidney injury (AKI) requiring renal replacement therapy (RRT). Despite progress in the management of AKI and high-quality catheter practices, vascular access remains the weak link in the chain of RRT and contributes to increased morbidity in hemodialysis patients, particularly through catheter dysfunction (stenosis and/or thrombosis) and infection [1, 2].

It is accepted practice to lock the lumen of non-tunneled hemodialysis catheters with an anticoagulant solution to prevent thrombosis, maintain catheter patency, and avoid infection between dialysis sessions. Heparin locks are considered the reference, but the use of heparin is associated with a number of complications, including inadvertent systemic administration potentially leading to coagulopathy and bleeding, heparin-induced thrombocytopenia [3], and allergic reactions [4], thus rendering heparin difficult to handle in the intensive care unit (ICU).

Antimicrobial locking solutions to reduce catheter-related infection (CRI) could be an attractive alternative, but must contain a high antimicrobial concentration to overcome the relative resistance of sessile bacteria in the catheter biofilm. Studies have shown that antibiotic locks decrease the risk of long-term hemodialysis infection [5], but, when used repeatedly, may promote the selection of resistant organisms [6, 7]. A randomized, double-blind, placebo-controlled trial comparing ethanol to saline solution failed to find a decrease in the frequency of infection of dialysis catheters in ICU patients [8].

Citrate locking solutions are a promising alternative to heparin. Citrate exerts its anticoagulant effect by its ability to chelate calcium, which is fundamental to the activation of the coagulation cascade, but also of platelet activity [9]. Randomized controlled trials (RCT) comparing heparin versus citrate lock solutions have been small [10–12] or used high concentrations of citrate, respectively, 30% [13] and 46.7% [14]. It is noteworthy that in 2000, the Food and Drug Administration (FDA) prohibited the use of citrate at concentrations greater than 4% due to the risk of metabolic disorders [15, 16], notably major hypocalcemia resulting in death in cases of systemic leakage [17].

The objective of this randomized, double-blind, multicenter, controlled trial was to compare the duration of event-free survival of the first non-tunneled hemodialysis catheter between trisodium citrate 4% and heparin as the catheter lock solution.

## Methods

### Study design

The study design has previously been published elsewhere [18]. Briefly, the VERROU-REA study was a randomized, prospective, multicenter, double-blind, controlled study designed to compare two strategies for locking non-tunneled hemodialysis catheters, namely trisodium citrate at 4% (Citra-Lock, Dirinco AG, Bern, Switzerland), versus unfractionated heparin (control group) at a concentration of 5000 IU/ml, used within the range of its currently approved indications for extracorporeal circulation and RRT. The study sponsor was the University Hospital of Dijon, France. An independent Data and Safety Monitoring Board (DSMB) monitored the safety of the trial and periodically assessed whether the trial should continue to planned termination. The study received approval for all participating centers from the local ethics committee (Comité de Protection des Personnes Est I) under the number 2013/14 and by the Agence National de Sécurité des Médicaments et des Produits de Santé (ANSM, French National Agency for the Safety of Medical Product and Devices, approval number 2013-000414-37). The first author drafted the manuscript, which was reviewed by the trial steering committee. Statistical analyses were performed in accordance with the International Conference on Harmonization Good Clinical Practice guidelines by the study statistician (AB). The authors attest that the study was performed in accordance with the protocol and vouch for the accuracy and completeness of the reported analyses.

### Study population

Patients were eligible for enrollment if they were aged 18 years or older, admitted to the ICU, with AKI requiring RRT, and in whom a first non-tunneled hemodialysis catheter was to be inserted by the jugular or femoral vein, provided informed consent, and had social security coverage.

Exclusion criteria were as follows:

- Catheters inserted by the subclavian approach.
- Contraindication to systemic anticoagulation (active uncontrolled bleeding, acute liver failure (factor V < 30%), thrombocytopenia < 30,000/mm^3^ in the absence of planned correction), documented or suspected heparin-induced thrombocytopenia. HIT was suspected in the presence of platelet count <100 000 g/l and/or a decrease of > 40% over baseline, occurring within a compatible timeframe after the introduction of heparin (5–8 days), and documented by immunoenzymatic tests (ELISA).
- Known allergy to citrate or heparin.
- Patients on chronic dialysis.
- Documented systemic bacterial infection not under treatment at the time of randomization.
- Patients not affiliated with a health insurance system (beneficiary or dependent).
- Pregnant patients.
- Patients with advance directives issued expressing the desire not to be resuscitated.
- Patient under tutorship or curatorship or judicial protection.
- Enrollment in any concomitant randomized trial with the same outcomes as VERROU-REA.

Written informed consent was obtained from the patient or responsible surrogate (see Additional file 1 for details).

### Randomization

Randomization was performed after verification of the inclusion and exclusion criteria via an online request using Tenalea^®^ software (Formsvision BV, Abcoude, The Netherlands). Allocation was based on a minimization technique taking into account the catheter insertion site (jugular or femoral), intended type of dialysis (continuous or intermittent) and Simplified Acute Physiology Score (< 55 or ≥ 55) [19]. Patients were assigned to treatment groups (citrate or heparin) using block randomization stratified by center. Patients were randomly assigned to one of the two groups in a 1:1 ratio. The Inserm CIC-1432 Clinical Epidemiology Unit (Dijon, France) managed the data.

### Interventions

Trisodium citrate at 4% or heparin, according to the study group, was instilled into both lumens of the catheter to attain a total volume corresponding to the volume of each branch. To preserve the blinding for the investigator, the nurse prepared the lock solution (see the Additional file 1 for details). Before each administration of citrate or heparin, the catheter lumen was flushed with 10 mL of saline as quickly as possible. Then, the lock solution (citrate or heparin) was injected slowly (over at least 10-s duration) into each lumen. Before initiation of RRT and use of the catheter, a minimum of 5 mL of liquid was extracted from each lumen. Catheter patency was verified by performing a blood return with a 20-mL syringe. All catheters had a minimum diameter of 13.5 French. All catheters were double-lumen catheters, 15 cm long for the right jugular route, 20 cm for the left jugular route, and 24 cm for the femoral route. Only one catheter per patient (i.e., the first inserted) was considered for analysis. Maximum barrier precautions were followed for catheter placement and manipulation [20]. The decision to use ultrasound guidance for catheter insertion was at the discretion of the operator. Patients were followed up for the duration of their hospital stay.

All staff likely to be involved in the management of patients included in the current study attended a dedicated session to undergo training in the monitoring procedures, and posters outlining the main procedures to remember were on permanent display in all participating departments. Details of the preparation of catheter locks at the pharmacy and patient management are described in Additional file 1.

### Study outcomes

The primary endpoint was the duration of event-free survival of the first non-tunneled hemodialysis catheter, defined as the time (in days) from catheter insertion to withdrawal, whatever the reason (infection, thrombosis, leakage or deteriorated catheter, intentional or accidental catheter removal, end of treatment, or death, whichever occurred first), up to 28 days. The secondary outcomes were as follows: the number of patients undergoing fibrinolysis for the first hemodialysis catheter, and the incidence rate of fibrinolysis per 1000 catheter-days, the incidence of catheter dysfunction per 1000 catheter-days; the incidence of catheter-related infection (CRI) and catheter-related bloodstream infections (CRBSI) per 1000 catheter-days; number of hemorrhagic events requiring transfusion of at least two units of packed red blood cells, length of stay in intensive or critical care, length of hospital stay, death rate at 28 days. A clinical event committee comprising two physicians (one hygiene specialist from the Department of Epidemiology and Hospital Hygiene and one infectious diseases specialist from the Department of Infectiology, University Hospital of Dijon, France), blinded to the treatment allocation, independently analyzed data and adjudicated all events as to the presence or not of catheter infection. All serious adverse events were adjudicated by a Central Pharmacovigilance Department (University Hospital of Dijon-Bourgogne, France) as to whether the event was attributable to the catheter lock. The definitions used for the outcomes are described in detail in Additional file 1.

### Statistical analysis

On the basis of published data at the time the trial was being designed [14, 21] and considering the survival duration of the first non-tunneled dialysis catheter as a time to event outcome, the prudent hypothesis of a median of 12 days in the citrate group versus 9 days in the heparin group was retained. We estimated that 386 patients (193 per group) were required to ensure 80% power at a bilateral alpha risk of 0.05, assuming a rate of 5% non-evaluable cases. An interim analysis was planned after inclusion of 50% of the patients (for details, see Additional file 1).

Quantitative variables are described as mean ± standard deviation (SD) when normally distributed, or as median (IQR) if non-normally distributed, and qualitative variables as number (percentage). The primary analysis was on an intention-to-treat basis. The duration of event-free survival of the first non-tunneled dialysis catheter, defined as the time from catheter insertion to withdrawal, whatever the reason, was compared between groups using the log-rank test, and the corresponding survival probabilities were charted using the Kaplan–Meier method. The time of withdrawal was not observed if the patient was discharged from the ICU to another unit with the first catheter in place. In this case, the time of withdrawal was censored at the time of discharge from the ICU. Sensitivity analysis was performed considering as events only catheter withdrawal due to lock failure (suspected infection, thrombosis, bleeding, leakage, or catheter dysfunction) and considering death, discontinuation of RRT, and other reasons for catheter withdrawal as competing events using a Fine–Gray model.

In secondary analyses, overall survival, 28-day mortality rate, in-ICU and in-hospital death, length of stay in the ICU, length of hospital stay and the incidence of catheter dysfunction per 1000 catheter-days, the incidence of CRI per 1000 catheter-days, the number of heparin-induced thrombocytopenia as well as hemorrhagic events requiring transfusion of at least two units of packed red blood cells were compared using the log-rank, Chi-square, Fisher’s exact, or Mann–Whitney *U* tests, as appropriate.

For per-protocol analysis, the same analyses were performed on patients grouped according to the treatment actually received.

All analyses were performed with SAS software, version 9.4 (SAS Institute Inc., Cary, NC, USA). The significance level was set at 0.05 for all final analyses.

## Results

### Patient population

**Fig. 1:**
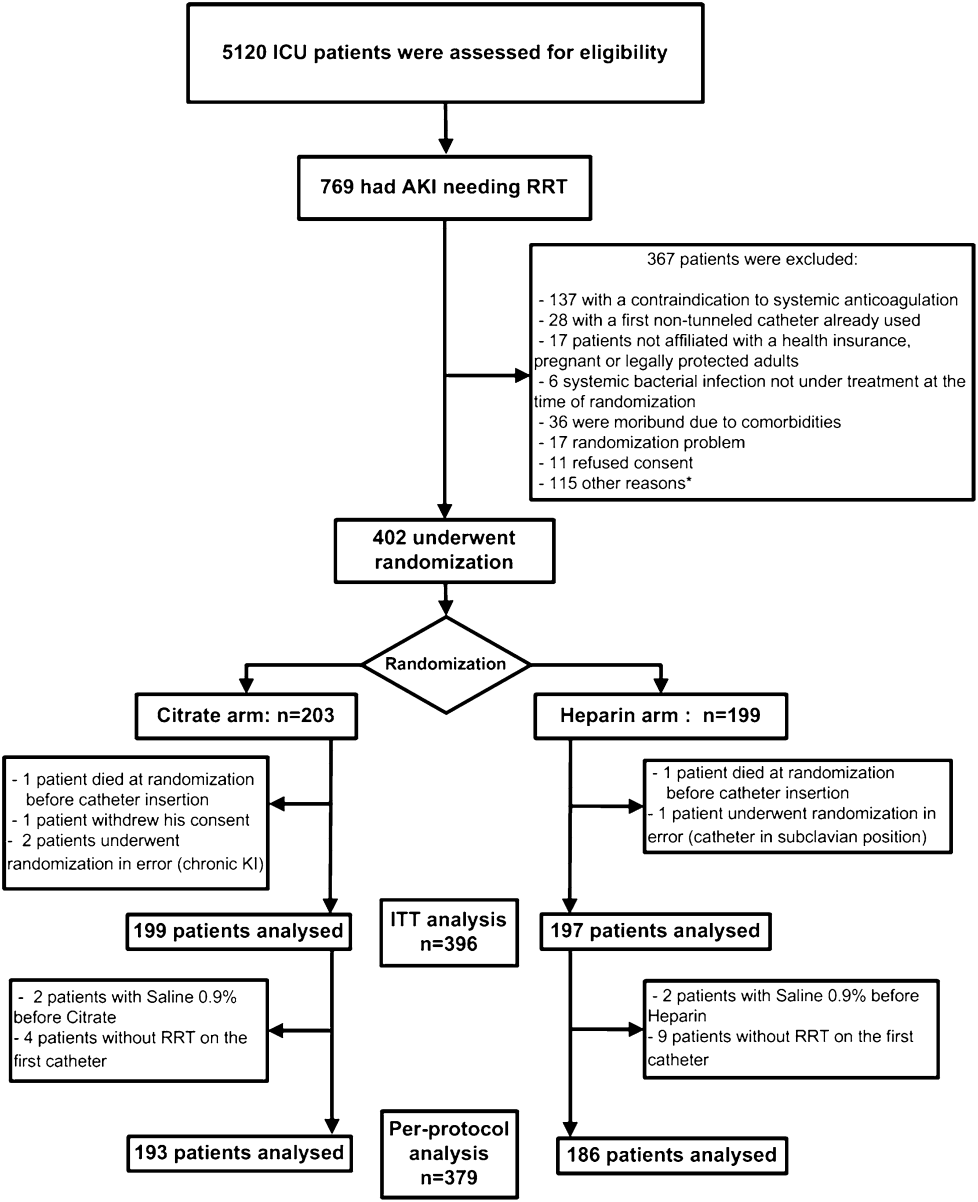
Flowchart of the VERROU-REA study population (*N* = 396)

The study was conducted between June 2013 and January 2016 in nine ICUs (seven university teaching hospitals and two general hospitals) in France. Among 402 randomized patients, 396 completed the trial. The flowchart of the VERROU-REA study population is shown in Fig. 1. By intention-to-treat, 199 patients were analyzed to the citrate group (treatment group) and 197 in the heparin group (control group).

There were no significant differences in baseline characteristics between groups (Table 1).

**Table 1:** Characteristics of the study population of the VERROU-REA study (*N* = 396)

Characteristics	Citrate (*N* = 199)		Heparin (*N* = 197)	
Age, years, mean (± SD)	69.4	(± 13.4)	69.5	(± 12.9)
Male gender, *n* (%)	127	(64%)	123	(62%)
BMI, mean (± SD)	30	(± 8.7)	29.8	(± 8.6)
Main reason for ICU admission, *n* (%)				
Neurologic	9	(5%)	6	(3%)
Cardiac	22	(11%)	30	(15%)
Renal	44	(22%)	29	(15%)
Respiratory	46	(23%)	41	(21%)
Sepsis or septic shock	63	(32%)	65	(34%)
Other shock	6	(3%)	11	(6%)
Other reasons	9	(5%)	14	(7%)
Charlson comorbidities score, mean (± SD)	6.1	(± 3.4)	5.9	(± 2.7)
SAPS II at ICU admission, mean (± SD)	62.5	(± 17.3)	63.0	(± 18.6)
SOFA at randomization, mean (± SD)	10.3	(± 3.6)	10.4	(± 3.5)
First catheter insertion site, *n* (%)				
Left internal jugular	16	(8%)	23	(12%)
Right internal jugular	61	(31%)	64	(32%)
Left femoral	43	(21%)	44	(22%)
Right femoral	79	(40%)	66	(34%)
Type of RRT for the first non-tunneled catheter, *n* (%)				
Both continuous and intermittent	36	(18%)	39	(20%)
Intermittent only	83	(42%)	82	(42%)
Continuous only	76	(38%)	67	(34%)
RRT not initiated^a^	4	(2%)	9	(5%)
Systemic anticoagulation, *n* (%)	119	(68%)	117	(66%)
Systemic antimicrobials for the first non-tunneled hemodialysis catheter, *n* (%)	160	(81%)	168	(85%)

SD, standard deviation; BMI, body mass index (kg/m^2^); ICU, intensive care unit; SAPS II, Simplified Acute Physiology Score II; SOFA, Sequential Organ Failure Assessment; Q1, Q3, first and third quartiles; RRT, renal replacement therapy; LMWH, low molecular weight heparin

^a^Reasons are detailed in the Additional file 1

The total median number of locks used for the first non-tunneled hemodialysis catheter was not statistically different between groups: 2 (IQR 1, 4) and 2 (IQR 1, 3) in the citrate and heparin groups, respectively.

### Primary and secondary outcomes (Table 2, Fig. 2)

**Fig. 2:**
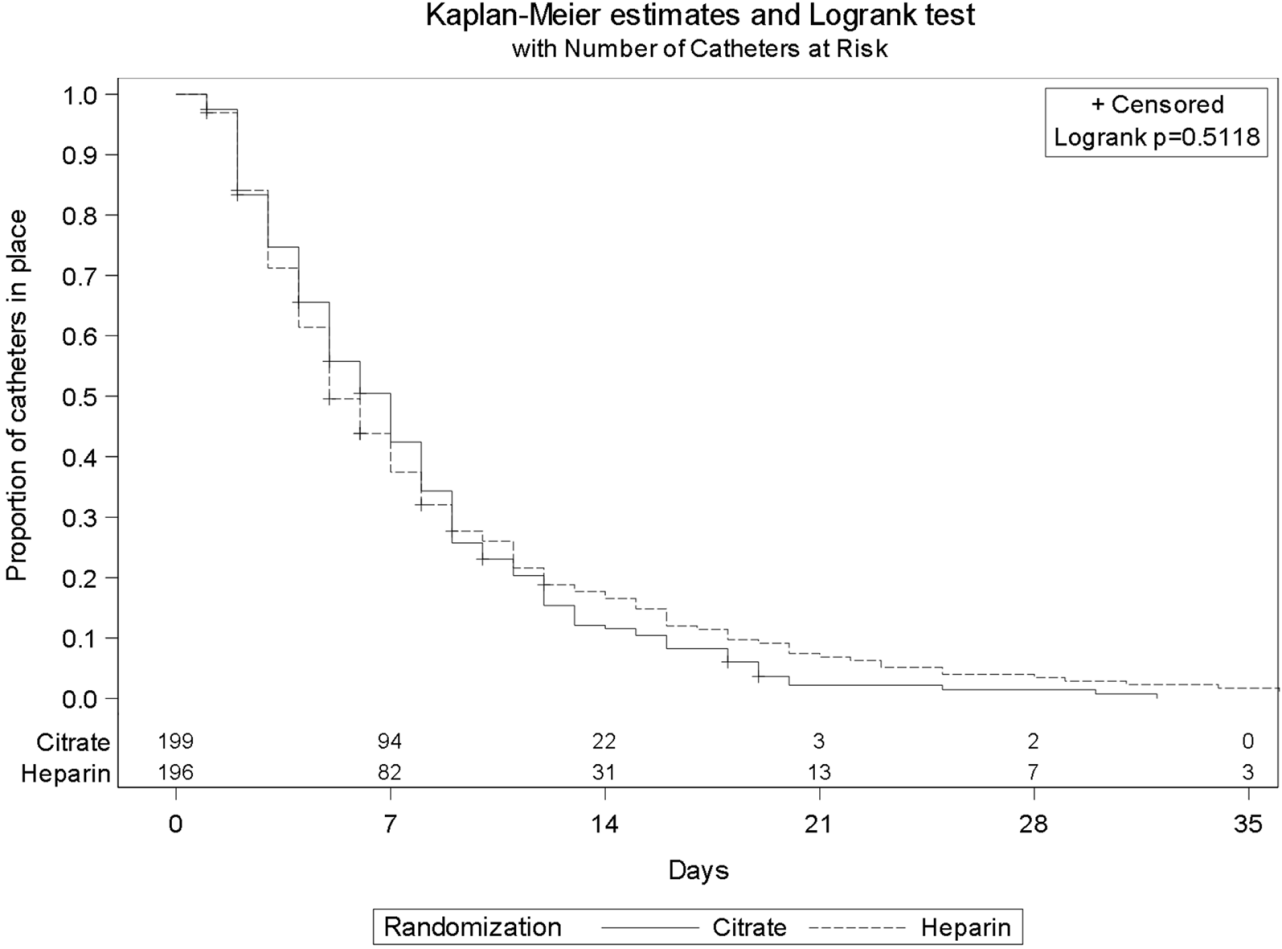
Event-free survival of the first non-tunneled hemodialysis catheter in the citrate and heparin groups

The duration of event-free survival of the first non-tunneled hemodialysis catheter was not significantly different between groups: 7 days (IQR 3–10) in the citrate group and 5 days (IQR 3–11) in the heparin group (*p* = 0.51) (Table 2, Fig. 2). Per-protocol analysis (*n* = 379 patients) yielded similar results (*p* = 0.42). Similarly, by sensitivity analysis, there was no significant difference in the time to withdrawal of the first catheter due to lock failure, considering other causes of withdrawal as competing events (*p* = 0.22). The reasons for catheter withdrawal are detailed in Table 3.

**Table 2:** Primary and Secondary Outcomes in the VERROU-REA Study (*N* = 396)

Outcome	Citrate (*N* = 199)	Heparin (*N* = 197)	*p* value
Overall duration of the first catheter (days), median (Q1, Q3)^a^	7 (3, 10)	5 (3, 11)	0.51
First hemodialysis non-tunneled catheter-days, *n*	1461	1590	
Fibrinolysis for the first non-tunneled hemodialysis catheter, *n* (%)	2 (1%)	0	1
Incidence rate per 1000 catheter-days	1.37	0	
Bleeding at the insertion site of the catheter, *n* (%)	1 (1%)	5 (3%)	0.12
Incidence rate per 1000 catheter-days	0.68	3.14	
Hematoma at the insertion site of the first catheter, *n* (%)	3 (2%)	1 (1%)	0.62
Incidence rate per 1000 catheter-days	2.05	0.63	
Thrombosis of the first non-tunneled hemodialysis catheter, *n* (%)	9 (5%)	3 (2%)	0.14
Incidence rate per 1000 catheter-days	6.16	1.89	
Local first catheter-related infection, *n* (%)	7 (14%)	8 (19%)	0.55
Incidence rate per 1000 catheter-days	4.79	5.03	
General first catheter-related infection, *n* (%)	4 (8%)	1 (2%)	0.37
Incidence rate per 1000 catheter-days	2.74	0.63	
First catheter-related bloodstream infection, *n* (%)	1 (2%)	0	1
Incidence rate per 1000 catheter-days	0.68	0	
Heparin-induced thrombocytopenia	4 (2%)	2 (1%)	1
Bleeding events during follow-up	41 (21%)	37 (19%)	0.65
Requiring transfusion packs red blood cells	22 (11%)	27 (14%)	0.42
Requiring transfusion of ≥ 2 packs red blood cells	20 (10%)	26 (13%)	0.33
Death at 28 days, *n* (%)	88 (44%)	94 (48%)	0.49
Death in ICU, *n* (%)	84 (42%)	92 (47%)	0.37
Length of ICU stay (from randomization) (days), median (Q1, Q3)	6 (3, 14)	6 (3, 12)	0.70
In-hospital death, *n* (%)	102 (51%)	108 (55%)	0.48
Length of hospital stay (from randomization) (days), median (Q1, Q3)	15 (6, 31)	12 (4, 27)	0.18

^a^Kaplan–Meier estimation and log-rank test with one missing data in the Heparin arm

**Table 3:** Reason for the withdrawal of the first non-tunneled hemodialysis catheter (among patients in whom withdrawal was observed)

Reason for withdrawal	Citrate (*N* = 199)	Heparin (*N* = 197)
Catheter withdrawal not observed	11 (6)	8 (4)
Reason for removal (if withdrawal observed), *n* (%)	188 (94)	189 (96)
Missing data	1 (0.5)	2 (1)
RRT stopped due to spontaneous recovery of renal function	62 (33)	69 (36.6)
Puncture site bleeding	0	2 (1)
Thrombosis catheter	5 (2.6)	2 (1)
Catheter dysfunction (inability to achieve and maintain a blood flow >200 mL/min)	28 (15)	18 (9.5)
Suspected catheter infection	12 (6.4)	12 (6.4)
Death	64 (34)	69 (36.6)
Other reasons	16 (8.5)	15 (7.9)
Accidental withdrawal of the catheter by the patient	4	2
Implantation of ECMO at the site of the dialysis catheter	2	0
Transfer to another hospital	5	7
Change of catheter site to allow the patient to get out of bed	2	3
Implantation of tunneled catheter	2	2
Decision to withdraw life support therapy	1	1

A total of five episodes of general first CRI and 15 episodes of local first CRI were observed, without statistically significant difference between the two groups. One episode of CRBSI was observed in the citrate group and none in the heparin group.

Thrombosis of the first catheter was observed in nine patients in the citrate group versus three in the heparin group (*p* = 0.14), requiring fibrinolysis in two patients in the citrate group.

Adverse events were not statistically different between groups. Among the cases of heparin-induced thrombocytopenia observed, none was related to the catheter lock. Among the bleeding events observed, 10/20 (50%) in the citrate group were associated with the catheter lock and 12/26 (46%) in the heparin group. The length of ICU stay, the length of hospital stay, and in-hospital and 28-day mortality rates were not statistically different between groups.

## Discussion

The main finding of this study is that in a first non-tunneled hemodialysis catheter, there was no significant difference in the duration of event-free survival of the catheter between trisodium citrate 4% and heparin as a locking solution. Catheter thrombosis, CRI, and adverse events were not statistically different between the two groups.

Studies comparing citrate with heparin as locks for non-tunneled hemodialysis catheters have generally been small and not adequately powered to provide conclusive evidence of safety and efficacy [10–14]. Data mainly concern patients undergoing chronic RRT with long-term tunneled catheters. In these patients, trisodium citrate 4% is considered as the reference lock solution, in order to prevent catheter dysfunction and infection [22, 23], although antimicrobial-containing citrate lock was reported to perform better than a heparin lock in the prevention of catheter-related infection in a meta-analysis of 13 randomized trials [13]. Conversely, heparin is considered as the reference for locking non-tunneled hemodialysis catheters, but there is insufficient evidence in the literature regarding its safety and efficacy in critically ill patients. Despite the paucity of data, the American Society of Diagnostic and Interventional Nephrology (ASDIN) and European Renal Best Practice (ERBP) consider 4% citrate and heparin to be acceptable alternatives for locking central venous catheters [22, 23]. Thus, it would appear logical that citrate could hold promise for the patient population of our study, notably for those with a contraindication to heparin.

To the best of our knowledge, only one randomized study, which included 291 patients in ten dialysis units, has previously compared the safety and efficacy of heparin versus citrate as a locking solution for non-tunneled catheters in hemodialysis patients [14]. The risk of catheter dysfunction was significantly lower in the citrate group, and the authors also noted a lower incidence of CRI in the citrate group as compared to the heparin group. Furthermore, the risk of bleeding and death from CRBSI was significantly lower in the citrate group. Indeed, it should be noted that this study was prematurely interrupted because of a significant difference in CRBSI between groups, in favor of the citrate group. We failed to observe any significant difference in CRBSI in our study. This could be explained by the fact that in the study by Weijmer et al., the citrate concentration used was 30% (instead of 4%), and critically ill patients were excluded. To explain these results, the authors rely on in vitro studies that demonstrated bactericidal and sporicidal activity of citrate (23% or 4%) and prevention of biofilm formation [24]. This is in contrast to heparin, which may in fact promote biofilm formation and increase the risk of infection [25]. A further potential explanation for the difference with our findings is that in our study, infectious events were adjudicated by a clinical events committee, and therefore, only confirmed catheter-related events are counted.

In a prospective quasi-experimental study using 46.7% citrate lock [26], Parienti et al. reported that the risk of CRI was not significantly different in the citrate group compared to either saline solution or heparin. Moreover, the mean duration of non-tunneled hemodialysis catheters was not statistically different between groups, as in our study. On the other hand, the use of citrate 46.7% in Parienti’s study was associated with less catheter dysfunction, in line with the higher rate of catheter dysfunction found in the saline solution group as compared to the citrate group by Hermite and colleagues in their study [21].

In a recent systematic review and meta-analysis [27], Grudzinski et al. compared the benefits and harms of citrate locking solutions versus heparin for non-tunneled hemodialysis catheters. The rates of death and CRBSI tended to be lower with citrate, but pooled effect estimates were not statistically significant. No significant differences in catheter exchange/replacement, thrombolysis, or all-cause hospitalization were found between groups in any of the pooled analyses. Conversely, citrate locking solutions were associated with significantly fewer bleeding episodes. In our VERROU-REA study, the number of bleeding episodes was not statistically different between the citrate and heparin groups, probably because patients at risk of hemorrhage were excluded from this study. Also, systemic anticoagulation was used in 68% in the citrate group and 66% in the heparin group, and thus, any effect of heparin would be subsumed by the overall effect of the anticoagulant therapy. Similarly, we failed to observe any difference in general or local infections, or in CRBSI between groups, probably because systemic antimicrobials were used while the first hemodialysis catheter was in place in, respectively, 81% and 85% in citrate and heparin groups. Furthermore, the study was not powered to detect a significant difference in these secondary endpoints.

Our study has several limitations. First, no analysis of the costs related to the use of citrate as a catheter lock solution (such as cost-effectiveness) was performed or planned. However, no such analysis was planned because in the French healthcare system, neither of these two catheter lock solutions is significantly more expensive than the other.

Second, our study population comprised mainly critically ill patients with sepsis and with a high rate of use of systemic anticoagulation and antibiotics, and thus, our findings cannot be generalized to other clinical situations. In addition, patients with a contraindication to anticoagulant therapy were excluded. Finally, the duration of the first catheter may have been too short to show a significant difference between groups, and in both groups, a median of only two locks was observed. Indeed, the sample size was calculated on the assumption that catheter survival would be 12 days in the citrate (intervention) group compared to 9 days in the heparin (control) group, based on previous publications. Unfortunately, in reality, by 7 days, more than 50% of the catheters had already been removed in both arms of the study, and largely due to events that were not the primary endpoint. Further studies are warranted that measure survival of subsequent catheters, and not just the first.

## Conclusion

In conclusion, our study did not show a significant difference between trisodium citrate 4% and heparin, as a catheter lock solution, in terms of the survival duration of a first non-tunneled hemodialysis catheter in critically ill patients. However, these findings should be interpreted in light of the study limitations and deserve to be confirmed in further studies that investigate catheter survival beyond the first catheter.

## Additional file

**Additional file 1.** Online supplement containing additional information relating to the methods, as well as author contributions and the full list of co-investigators in the VERROU-REA Study group.

## Data Availability

Data are available on reasonable request to the corresponding author.
