# Supplementary material for: Trisodium citrate 4% versus heparin as a catheter lock for non-tunneled hemodialysis catheters in critically ill patients: a multicenter, randomized clinical trial
Source: Ann Intensive Care. 2019 Jul 1;9:75. doi: 10.1186/s13613-019-0553-4 (PMC6603108; doi:10.1186/s13613-019-0553-4)
Supplement: Supplementary file 1 — Additional file 1. Online supplement containing additional information relating to the methods, as well as author contributions and the full list of co-investigators in the VERROU-REA Study group. [file 13613_2019_553_MOESM1_ESM.docx]

**Trisodium citrate 4% vs heparin as a catheter lock for** **non-tunneled hemodialysis catheters in critically ill patients: a multicenter randomized clinical trial**

Jean-Pierre Quenot, MD, PhD., Julie Helms, MD, PhD, Abderrahmane Bourredjem, MSc, Auguste Dargent, MD, Ferhat Meziani, MD, PhD, Julio Badie, MD, Gilles Blasco, MD, Gaël Piton, MD, PhD, Gilles Capellier, MD, PhD, Chaouki Mezher, MD, Jean-Michel Rebibou, MD, PhD, Abdelouaid Nadji, MD, Thomas Crepin, MD, Saber Davide Barbar, MD, PhD, Camille Fleck, PharmD, Amélie Cransac, PharmD, Mathieu Boulin, Pharm D, PhD, Christine Binquet, MD, PhD, Agnès Soudry-Faure, PharmD, PhD , Rémi Bruyère, MD, and for the VERROU-REA Trial Investigators and the CRICS TRIGGERSEP Group (Clinical Research in Intensive Care and Sepsis Trial Group for Global Evaluation and Research in Sepsis)

**Online Data Supplement**

**TABLE OF CONTENTS OF SUPPLEMENTARY MATERIAL**

[1. List of co-investigators of VERROUREA study group 3](#_Toc11252713)

[2. Informed consent 5](#_Toc11252714)

[3. Preparation of catheter locks at the hospital pharmacy 6](#_Toc11252715)

[4. Details of the study intervention 7](#_Toc11252716)

[5. Outcome Definitions in the Verrou-Rea Study 8](#_Toc11252717)

[6. Sample size calculation and interim analysis 9](#_Toc11252718)

[6.1. Sample Size Calculation 9](#_Toc11252719)

[6.2. Interim analyses 9](#_Toc11252720)

[8. Reasons for non-initiation of RRT with the first catheter 12](#_Toc11252721)

[9. Author Contributions 13](#_Toc11252722)

[10. References for Supplementary Material 14](#_Toc11252723)

# List of co-investigators of VERROUREA study group

| **Name** | **Title** | **Institute** | **Department** |
| --- | --- | --- | --- |
| ANDREU Pascal | MD | CHU de Dijon | Médecine Intensive-Réanimation |
| CHARLES Pierre-Emmanuel | MD, PhD | CHU de Dijon | Médecine Intensive-Réanimation |
| PRIN Sébastien | MD | CHU de Dijon | Médecine Intensive-Réanimation |
| LARGE Audrey | MD | CHU de Dijon | Médecine Intensive-Réanimation |
| TOITOT Amaury | MD | CHU de Dijon | Médecine Intensive-Réanimation |
| ROUDAUT Jean-Baptiste | MD | CHU de Dijon | Médecine Intensive-Réanimation |
| HAMET Maël | MD | CHU de Dijon | Médecine Intensive-Réanimation |
| JACQUIOT Nicolas | MD | CHU de Dijon | Médecine Intensive-Réanimation |
| PAVON Arnaud | MD | CHU de Dijon | Médecine Intensive-Réanimation |
| BOUHEMAD Bélaïd | MD, PhD | CHU de Dijon | Département d’Anesthésie-Réanimation |
| COMBES Jean-Christophe | MD | CHU de Dijon | Réanimation Neuro-Traumatologique |
| MIREK Sébastien | MD | CHU de Dijon | Réanimation Neuro-Traumatologique |
| ZANETTA Gilbert | MD | CHU de Dijon | Soins Intensifs Néphrologie |
| TORNER Stéphane | MD | CHU de Dijon | Soins Intensifs Néphrologie |
| ROUCHE Jonathan | MD | CHU de Dijon | Soins Intensifs Néphrologie |
| VIVET Bérengère | MD | CHU de Besançon | Réanimation Médicale |
| KUMMERIEN Christine | MD | CHRU de Strasbourg | Réanimation Médicale |
| RAHMANI Hassène | MD | CHRU de Strasbourg | Réanimation Médicale |
| RABOUËL Yannick | MD | CHRU de Strasbourg | Réanimation Médicale |
| CLERE-JEHL Raphaël | MD | CHRU de Strasbourg | Réanimation Médicale |
| SAMAIN Emmanuel | MD, PhD | CHU de Besançon | Réanimation Chirurgicale |
| PILI-FLOURY Sébastien | MD, PhD | CHU de Besançon | Réanimation Chirurgicale |

# Informed consent

When patients met the inclusion criteria, and did not present any non-inclusion criteria, they were informed (orally, with supporting documentation in written format) about the study by the investigators, and invited to participate. If patients were temporarily incapable of receiving the appropriate information or making an informed decision regarding consent to participate, they could still be included if informed consent was given by the patient’s surrogate or legal representative. Consent was obtained if patients subsequently regained the capacity to understand the study procedures and provide informed consent. Patients could be included in emergency situations if their condition precluded consent and no legal representative or close relative was available to provide consent. In this case, the investigator noted and justified in the patient’s medical record that the patient was temporarily unable to receive the study information and provide informed consent, and that no legal representative or close relative could be reached. Written consent was subsequently obtained from patients, as soon as their clinical status allowed.

# Preparation of catheter locks at the hospital pharmacy

Both catheter lock solutions used in the study were prepared by the pharmacy of Dijon University Hospital and provided to the pharmacy of the participating hospitals, with specific labels identifying the study, and in their original packaging, for storage in each participating department in the usual conditions according to the treatment attributed by randomization. The presentation of both solutions (heparin and citrate) was identical (5 mL syringe). Special labels indicating the regulatory information relating to clinical trials were provided by the pharmacy of the University Hospital of Dijon and stuck to the syringe by the nurse who prepared the catheter lock solution.

Reception, dispensing and return of study drugs was documented in writing in a dedicated study file kept in the pharmacy. Units of unused study drug were returned to the pharmacy by the investigator after withdrawal of the first catheter. Since the study was a double-blind design, the investigators are unaware of the type of lock solution being instilled in the catheter for each patient. To preserve the blinding, the lock solution was provided in a box. The preparation to be injected as a catheter lock was prepared in a 5 mL syringe by a nurse from the unit, in isolation, in a specific room dedicated to the study procedures. The nurse then provided the pre-prepared syringe to the physician in charge of the patient’s management, who injected the lock solution slowly into the catheter lumen. In order to keep the blinding intact, the nurse who prepared the syringe with the catheter lock was NOT in charge of the patient’s management.

# Details of the study intervention

All staff likely to be involved in the management of patients included in the current study attended a dedicated session to undergo training in the monitoring procedures, and posters outlining the main procedures to remember were on permanent display in all participating departments.

**At the time of insertion of the first hemodialysis catheter**, the catheter lumens were flushed with 10 mL of saline as quickly as possible. Then, the lock solution (heparin or citrate) was injected slowly (over at least 10 seconds duration) into each lumen, to attain a total volume corresponding to the volume (in mL) of each branch of the catheter according to the manufacturer’s instructions. In emergency situations, the patient could be connected to the dialysis machine directly, then randomization was performed for attribution of one of the two catheter locks to be instilled after disconnection from the dialysis machine.

**Immediately before initiation of RRT and use of the catheter**, a minimum of 5 mL of liquid was extracted from each lumen (volume greater than the contents of each lumen) to ensure that the catheter lock solution as not released into the circulation.

**At the end of RRT,** 10 mL of saline solution was instilled into each branch of the catheter as quickly as possible. Then, the lock solution (heparin or citrate) was injected slowly (over at least 10 seconds duration) into each lumen, to attain a total volume corresponding to the volume (in mL) of each branch of the catheter according to the manufacturer’s instructions.

The catheter was used for dialysis only. It was forbidden to use it for infusion of other solutions or medications, even for a short duration.

The lock solution was left in place between RRT sessions, including during transfer for imaging or surgery, for example.

All catheters had a minimum diameter of 13.5 French. All catheters were double-lumen catheters, 15 cm long for the right jugular route, 20 cm for the left jugular route, and 24 cm for the femoral route. Only one catheter per patient (i.e. the first inserted) was considered for analysis.

# Outcome Definitions in the Verrou-Rea Study

| Catheter dysfunction: Catheter dysfunction is defined as the inability to achieve and maintain a blood flow >200 mL/min despite changing the patient’s position, inverting the lines, and flushing with saline solution.  Catheter thrombosis: Catheter thrombosis was defined as the inability to maintain extra-corporeal circulation (for reasons other than mechanical, such as a kinked catheter, or hypovolemia) and/or the presence of thrombus observed during manipulation of the catheter with the syringe (e.g. during catheter purge) and/or the presence of thrombus observed at catheter withdrawal.  Catheter thrombosis will be treated by the injection of 5,000 to 10,000 IU/mL of urokinase in each lumen of the catheter with a volume equivalent to that of the catheter (noted on each catheter). After 15 minutes, the urokinase will be aspirated with a syringe and dialysis will resume. If urokinase treatment fails to resolve catheter thrombosis, the catheter will be changed. |
| --- |
| Cather-Related Infections (CRI) include the following:  Local catheter-related infection, defined as a positive catheter-tip culture ≥10^3^ CFU/ml with presence of pus or oozing at the catheter insertion site, or tunnelitis.  General catheter-related infection, defined as a positive catheter-tip culture ≥10^3^ CFU/ml with disappearance of general signs of infection within 48 hours after catheter withdrawal.  Catheter-Related Blood Stream Infection (CRBSI), defined as the presence of fever (temperature>38°C) and positive blood culture taken from the dialysis catheter and a peripheral line in the absence of any other source of infection, with a central-to-peripheral quantitative blood culture ratio >5 or a differential period of central line culture versus peripheral blood culture positivity > 2 hours, with central hemoculture showing a positive result more quickly than peripheral culture.  Systematic samples were performed at the puncture site in case of any change in skin colour (e.g. redness) and/or presence of pus or oozing suggestive of infection. All samples were analysed by the bacteriology laboratory. At catheter removal, catheter tips were cultured using a simplified quantitative broth dilution technique with vortexing. If infection is confirmed, antibiotic therapy will be initiated.  The same microorganism had to be identified from central and peripheral blood cultures for a diagnosis of CRI.  A clinical event committee comprising two physicians (Dr. Aho, from the Department of Epidemiology and Hospital Hygiene, University Hospital of Dijon, and Prof. Piroth, from the Department of Infectiology, University Hospital of Dijon, France) blinded to the treatment allocation will independently analyse data and adjudicate as to the presence or not of catheter infection. |

# Sample size calculation and interim analysis

## Sample Size Calculation

The sample size was calculated using PASS software (version 11, Kayesville, Utah, USA), based on the following assumptions: The study by Hermite et al (1) compared the event-free survival of catheters with citrate locks versus saline solution in ICU patients with an indication for RRT. In this study, the median duration of catheter survival with citrate locking solution was 12 days [Interquartile range, IQR 8-17], versus 6 days [3-10] for catheters locked with saline solution (hazard ratio (HR) 2.12, 95% confidence interval (CI) 1.32-3.4), p=0.0019). The study by Weijmer et al (2) reported a relative risk (RR) of 0.69 (95%CI 0.44-1.08), p=0.11) for premature withdrawal of non-tunneled, uncuffed catheters with citrate versus heparin. On the basis of these findings, in the prudent hypothesis of a reduction of 25% in the risk of catheter-related events (median of 12 days in the citrate group versus 9 days in the heparin group), at an alpha risk of 5% and a beta risk of 20%, a total of 366 patients (183 per group) was deemed to be required. Accounting for possible crossovers between groups (less than 5% switching from heparin to citrate) due to heparin-induced thrombocytopenia, and allowing for patients lost to follow-up, the final sample size was calculated as 386 patients (193 patients included per group).

## Interim analyses

One interim analysis was planned in this study. It was conducted by the statistician (AB) on June 19^th^, 2015, after 199 inclusions. The number of days with the first catheter in place was recorded in both groups and compared using a log-rank test with an alpha risk set at 0.01, according to the method proposed by Peto (3) so as not to change the level of significance of the final primary analysis. After this analysis, the DSMB recommended continuing the study.

The following table summarizes the number of deaths and the p-value from testing at the interim and final analysis:

|  | **Citrate** | | **Heparin** | | **p-value** |
| --- | --- | --- | --- | --- | --- |
| **Interim analysis** | N=99 |  | N=100 |  |  |
| Duration of first catheter (days), median (Q1, Q3) | 5 | (3, 9) | 5 | (3, 8.5) | 0.81 |
|  |  |  |  |  |  |
| **Final analysis** | N=199 |  | N=196 |  |  |
| Duration of first catheter (days), median (Q1, Q3) | 7 | (3, 10) | 5 | (3, 11) | 0.51 |

1. **Details of “Other Reasons” for Exclusion from the VERROU REA Study**

- No study investigator available (during night and weekend duty) (N=22)
- Workload too busy to allow randomization of the patient within the specified time window (N=19)
- No next of kin available to provide consent and patient decisionally incapacitated (patients under sedation and/or mechanical ventilation) (N=18)
- Patient included in another clinical trial (N=15)
- Emergency RRT and/or technical problems (N=14)
- Patient transferred to another unit (N=12)
- Patient already on dialysis at the time of potential inclusion (N=9)
- Screening forms illegible or missing data (n=6)

# Reasons for non-initiation of RRT with the first catheter

|  | **Citrate (N=4)** | | **Heparin (N=9)** | |
| --- | --- | --- | --- | --- |
| Spontaneous recovery of renal function | 2 | 50% | 3 | 33% |
| Death before initiation of RRT | 0 |  | 3 | 33% |
| Position and/or type of catheter inappropriate | 1 | 25% | 2 | 22% |
| Catheter dysfunction (inability to achieve and maintain a blood flow >200 mL/min) | 1 | 25% | 1 | 11% |

# Author Contributions

Study conception and design: JPQ, AD, SB, CB, ASF, RB.

Acquisition and analysis of data: JH, AB, AD, FM, JB, GB, GP, GC, CM, JMB, AN, TC, SB, CF, AC, BM, JPQ, RB.

Interpretation of data: JPQ, AB, AD, SB, CB, ASF, RB.

Drafting of the first draft of the manuscript: JPQ, AB, AD, SB, AC, CB, RB.

All authors approved the final submitted version and all authors have agreed both to be personally accountable for the author's own contributions and to ensure that questions related to the accuracy or integrity of any part of the work, even ones in which the author was not personally involved, are appropriately investigated, resolved, and the resolution documented in the literature.

The first author drafted the manuscript, which was reviewed by the trial steering committee. Statistical analyses were performed in accordance with the International Conference on Harmonization Good Clinical Practice guidelines by the study statistician (AB).

# References for Supplementary Material

1. Hermite L, Quenot JP, Nadji A, Barbar SD, Charles PE, Hamet M, et al. Sodium citrate versus saline catheter locks for non-tunneled hemodialysis central venous catheters in critically ill adults: a randomized controlled trial. Intensive Care Med. 2012;38(2):279-85.

2. Weijmer MC, van den Dorpel MA, Van de Ven PJ, ter Wee PM, van Geelen JA, Groeneveld JO, et al. Randomized, clinical trial comparison of trisodium citrate 30% and heparin as catheter-locking solution in hemodialysis patients. J Am Soc Nephrol. 2005;16(9):2769-77.

3. Peto R, Pike MC, Armitage P, Breslow NE, Cox DR, Howard SV, et al. Design and analysis of randomized clinical trials requiring prolonged observation of each patient. I. Introduction and design. Br J Cancer. 1976;34(6):585-612.
